# Gut microbiota and sepsis and sepsis-related death: a Mendelian randomization investigation

**DOI:** 10.3389/fimmu.2024.1266230

**Published:** 2024-01-31

**Authors:** Weifeng Shang, Sheng Zhang, Hang Qian, Sisi Huang, Hui Li, Jiao Liu, Dechang Chen

**Affiliations:** Department of Critical Care Medicine, Ruijin Hospital, Shanghai Jiao Tong University School of Medicine, Shanghai, China

**Keywords:** gut microbiota, sepsis, sepsis-related death, causality, Mendelian randomization

## Abstract

**Background:**

It is unclear what the causal relationship is between the gut microbiota and sepsis. Therefore, we employed Mendelian randomization (MR) to determine whether a causal link exists between the two.

**Methods:**

This study used publicly available genome-wide association studies (GWAS) summary data of gut microbiota, sepsis, sepsis (critical care), and sepsis (28-day death in critical care) to perform a two-sample MR analysis. To ensure the robustness of the results, we also conducted a sensitivity analysis.

**Results:**

For sepsis susceptibility, inverse variance weighted (IVW) estimates revealed that *Victivallales* (OR = 0.86, 95% CI, 0.78–0.94, *p* = 0.0017) was protective against sepsis, while *Lentisphaerae* (OR = 0.89, 95% CI, 0.80–0.99), *Gammaproteobacteria* (OR = 1.37, 95% CI, 1.08–1.73), *Clostridiaceae1* (OR = 1.21, 95% CI, 1.04–1.40), *RuminococcaceaeUCG011* (OR = 1.10, 95% CI, 1.01–1.20), *Dialister* (OR = 0.85, 95% CI, 0.74–0.97), and *Coprococcus2* (OR = 0.81, 95% CI, 0.69–0.94) presented a suggestive association with the development of sepsis (all *p* < 0.05). For sepsis (critical care), IVW estimates indicated that *Lentisphaerae* (OR = 0.70, 95% CI, 0.53–0.93), *Victivallales* (OR = 0.67, 95% CI, 0.50–0.91), *Anaerostipes* (OR = 0.49, 95% CI, 0.31–0.76), *LachnospiraceaeUCG004* (OR = 0.51, 95% CI, 0.34–0.77), and *Coprococcus1* (OR = 0.66, 95% CI, 0.44–0.99) showed a suggestive negative correlation with sepsis (critical care) (all *p* < 0.05). For sepsis (28-day death in critical care), IVW estimates suggested that four bacterial taxa had a normally significant negative correlation with the risk of sepsis-related death, including *Victivallales* (OR = 0.54, 95% CI, 0.30–0.95), *Coprococcus2* (OR = 0.34, 95% CI, 0.14–0.83), *Ruminiclostridium6* (OR = 0.43, 95% CI, 0.22–0.83), and *Coprococcus1* (OR = 0.45, 95% CI, 0.21–0.97), while two bacterial taxa were normally significantly positively linked to the risk of sepsis-related death, namely, *Mollicutes* (OR = 2.03, 95% CI, 1.01–4.08) and *Bacteroidales* (OR = 2.65, 95% CI, 1.18–5.96) (all *p* < 0.05). The robustness of the above correlations was verified by additional sensitivity analyses.

**Conclusion:**

This MR research found that several gut microbiota taxa were causally linked to the risk of sepsis, sepsis in critical care, and sepsis-related 28-day mortality in critical care.

## Introduction

Sepsis is an organ dysfunction that is described as a life-threatening disorder deriving from a dysfunctional host response to infection. According to pertinent data, 30 million cases of sepsis occur annually worldwide, with a high mortality rate of 16% to 33% (1, 2). A key step in improving the prognosis of sepsis is early identification and treatment. Blood culture is frequently employed as the diagnostic standard for sepsis. However, it is time-consuming and has a poor positive rate (3). At present, no specific therapeutic drug exists for sepsis, which is managed through a combination of antibiotic therapy, organ protection, and fluid resuscitation. Hence, it is imperative to identify reliable early detection indicators as well as exact therapy targets for sepsis.

Gut microbiota is the collection of all microorganisms that colonize the gastrointestinal system and influence basic body functions such as digestion, energetic metabolism, and immune response (4). A growing number of studies have shown a link between gut microbiota dysbiosis and sepsis, which were mainly manifested as a decrease in beneficial bacteria and an increase in pathogenic bacteria (5–11). For example, Sun et al. discovered that the relative abundance of the *Proteobacteria* phylum and *Enterococcaceae* family was significantly elevated in sepsis patients compared to healthy controls, whereas the *Firmicutes* phylum and *Lachnospiraceae* family exhibited a relatively lower abundance (11). Furthermore, a retrospective cohort study of 10,996 participants by Prescott et al. suggested that intestinal microbial disturbances increased the risk of sepsis-related hospitalization and were associated with the severity of sepsis (7). Similar effects of gut microbiota exhaustion and loss of diversity to death rates have also been displayed in several sepsis experimental models (12, 13). However, the current associations were largely on the basis of observational studies with confounding factors, and the causality between gut microbiota and sepsis is not clear and requires more direct evidence.

Mendelian randomization (MR) is a type of instrumental variable (IV) analysis that employs genetic variations to determine the causality between exposure and outcome (14). The most common genetic variants are single-nucleotide polymorphisms (SNPs), which are randomly assigned to offspring along with gametes and occur before disease onset, and thus, MR is less susceptible to confounding factors and reverse causation. To date, despite several MR analyses investigating the causal relationship between gut microbiota and sepsis, the selection of genome-wide association studies (GWAS) data for sepsis outcomes is not entirely consistent (15, 16). The GWAS outcome data for sepsis selected by Chen et al. were derived from the 2020 UK Biobank (10,154 cases; 452,764 controls), and these are not the most up-to-date GWAS data available (15), while the study conducted by Zhang et al. incorporated various sepsis outcome GWAS data, which included patients below 75 years, 28-day mortality, critical care units (ICU), and 28-day mortality in ICU. However, they did not opt for the most recent sepsis outcome data available (16). Therefore, we chose to re-perform the MR analysis of gut flora and sepsis using the most comprehensive and up-to-date sepsis GWAS outcome data to investigate the causal relationship between gut microbiota and the risk of sepsis and sepsis-related death, elucidating the etiology of sepsis and suggesting innovative ideas for earlier diagnosis and treatment of this disease.

## Materials and methods

### Study design

**Figure 1:**
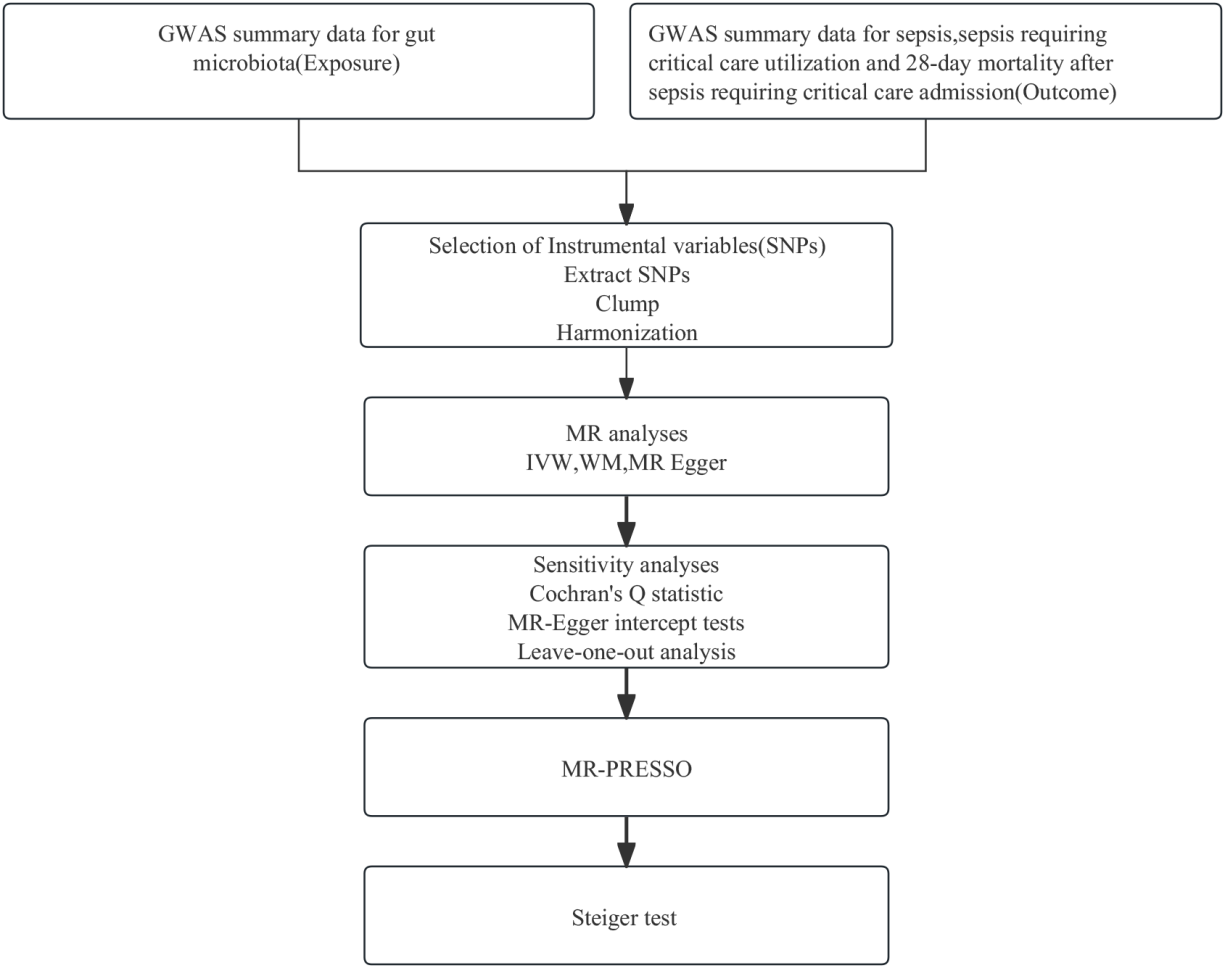
The analysis process of our research. GWAS, genome-wide association study; MR, Mendelian randomization; SNPs, single-nucleotide polymorphisms; IVW, inverse variance weighted; WM, weighted median; MR-PRESSO, Mendelian Randomization Pleiotropy RESidual Sum and Outlier.

Employing summary statistics derived from GWAS, we conducted two-sample MR analyses to evaluate the causality of the gut microbiota on the risk of sepsis, sepsis requiring critical care, and sepsis (28-day death in critical care). IVs are chosen to meet three assumptions: IVs are required to be strongly associated with the exposure of interest; IVs must be independent of unmeasured confounders; and IVs influence outcomes only through the exposure of interest (17). In this MR study, gut microbiota, and sepsis, sepsis (critical care), and sepsis (28-day death in critical care) were used as exposure and outcome, respectively. A flowchart presenting the whole procedure is shown in **Figure 1**. The present study used openly de-identified data from participant studies that were approved by the ethical standards committee for human experimentation. This study did not require separate ethical approval.

### Gut microbiota sample

Summary statistics pertaining to the human gut microbiota composition were derived from a comprehensive GWAS meta-analysis encompassing 24 cohorts derived from ethnically diverse backgrounds, including the United States, Canada, Israel, Korea, Germany, Denmark, the Netherlands, Belgium, Sweden, Finland, and the United Kingdom (*N* = 18,340). Among these, 20 cohorts included a sole representative sample, with the majority of participants being of European descent (16 cohorts, *N* = 13,226). A total of 122,110 variant sites for 211 taxa (9 phyla, 16 classes, 20 orders, 35 families, and 131 genera) were identified. Adjustments were made for sex, age, first 10 principal components and genotyping batch during the analysis (18). Summary-level statistics for the associated studies can be found on the website (https://mibiogen.gcc.rug.nl).

### Sepsis samples

Summary statistics for sepsis phenotypes were extracted from the IEU OpenGWAS, while summary-level data from the UK Biobank were also utilized (https://gwas.mrcieu.ac.uk/). The UK Biobank is an extensive cohort of UK adult participants; details were found in other sections (19). Sepsis phenotypes included sepsis (total cases: 11,643; total controls: 474,841), sepsis requiring critical care (total cases: 1,380; total controls: 429,985), and sepsis-related 28-day mortality in critical care (total cases: 347; total controls: 431,018). In the hospital episode statistics provided by the UK Biobank, cases were incorporated when the code was in the primary or secondary diagnostic category, adjusted for age, gender, microchip, and the first 10 principal components using regenie v2.2.4 (20). Sepsis admissions were identified by ICD codes from the UK Biobank linking secondary care data. In line with existing literature, ICD-10 codes A02, A39, A40, and A41 were used to identify sepsis (21). All study participants were of European ancestry.

### Selection of IVs

Five levels of assessment of bacterial taxa (phylum, order, order, family, and genus) were performed. As the eligible number of IVs was extremely small (*p* < 5×10^−8^), a relatively high threshold (*p* < 1×10^−5^) was used, which was in line with Ni et al. (22). In parallel, we performed quality control according to the following steps: First, the linkage disequilibrium (LD) threshold was set as 0.001, and the clumping window was 10 Mb. Second, we computed the *F*-statistic to quantify the genetic variation strength and then abandoned SNPs with an *F*-statistic of less than 10, indicating insufficient strength (23). Third, data were harmonized between gut microbiota and sepsis datasets, and SNPs with minor allele frequency (MAF)≤0.01, ambiguities, and palindromes were excluded. Ultimately, the gut microbiota linked to the outcome (*p* < 1.0×10^−5^) and gut microbiota with fewer than three SNPs were eliminated from the analysis. Following the above steps, the remaining SNPs were eventually used as IVs.

### Primary analysis

For the primary analysis, we employed random-effects inverse variance weighted (IVW) estimation, which combines the Wald ratio of each SNP to the outcome, assuming all genetic variations are valid. This approach offers the highest power for MR estimation, yet it is susceptible to multidirectional bias (24). Therefore, IVW was used as the primary method to estimate the cause and effect of gut microbiota on the risk of sepsis, sepsis requiring critical care, and sepsis-related 28-day mortality in critical care.

### Sensitivity analysis

Sensitivity analyses were subsequently performed to assess the bias of the MR assumptions for the identified significant estimates (*p*_IVW_ < 0.05). Some other MR analyses, such as weighted median and MR-Egger regression, were also used as complementary methods. MR-Egger regressions can test for multiplicity and considerable heterogeneity of imbalances, whereas for the same change in underexposure, larger sample sizes are required (25). In situations where at least half of the weighted variance provided by the horizontal pleated product effect is valid, the weighted median method offers consistent estimates of the effect (26). In addition, sensitivity analyses are also critical in MR studies to evaluate any bias of the MR assumptions. Consequently, Cochran’s *Q* statistic, MR-Egger intercept tests, and leave-one-out (LOO) analyses are employed to identify the presence of heterogeneity and pleiotropy, as well as evaluate the robustness of the obtained results. In particular, Mendelian Randomization Pleiotropy RESidual Sum and Outlier (MR-PRESSO) tests were conducted to test for outliers with potential horizontal pleiotropy. MR-PRESSO enables the estimation of SNP levels and overall heterogeneity, facilitating the detection of horizontal pleiotropy. In contrast, the outlier test comparing expected and observed variant distributions identifies outlier variants. Should any outliers be discovered, they are eliminated to produce unbiased causal estimates from the outlier-corrected MR analysis (27).

Hence, potential eligible candidate gut microbiome for participation in sepsis development was identified as follows: (1) *p*_IVW_ < 0.05; (2) the direction and amplitude of the three MR methods were consistent; (3) no heterogeneity or multiplicity was observed; and (4) no high-impact points were noted in the LOO analyses. The significant thresholds for each level were adjusted for multiple testing as follows: phylum *p* = 5.56 × 10^−3^ (0.05/9), class *p* = 3.13×10^−3^ (0.05/16), order *p* = 2.50 ×10^−3^ (0.05/20), family *p* = 1.43 × 10^−3^ (0.05/35), and genus *p* = 3.82 × 10^−4^ (0.05/131).

A nominal significant association was considered for *p*_IVW_ < 0.05 but exceeding the Bonferroni-corrected threshold. All analyses were run by using the R package TwoSampleMR (version 0.5.6) in R (version 4.1.1).

### Confounding analysis and Steiger test

Despite employing various statistical approaches in sensitivity analyses to investigate potential violations of MR assumptions, we also utilized the Phenoscanner V2 website (http://www.phenoscanner.medchsl.cam.ac.uk/) to examine whether gut microbial-related SNPs were concurrently associated with multiple common risk factors that might influence MR estimates, including sex (28), obesity (29), and diabetes (30). If the correlation between SNPs and these potential confounders reached a threshold value of *p* < 1×10^−5^, IVW was reiterated following the removal of these SNPs to validate the robustness of the findings. In addition, the MR Steiger test was performed on bacteria found to be causally related to sepsis, sepsis requiring critical care, and sepsis-related 28-day mortality in critical care, to verify the directionality of the results due to exposure, and *p* < 0.05 was considered statistically significant (31).

### Reporting guidelines

This study followed the guidelines of the Strengthening the Reporting of Observational Studies in Epidemiology Using MR (STROBE-MR), a checklist of which can be found in the Supporting Information (**Supplementary Table S1**) (32).

## Results

### Sepsis risk

**Figure 2:**
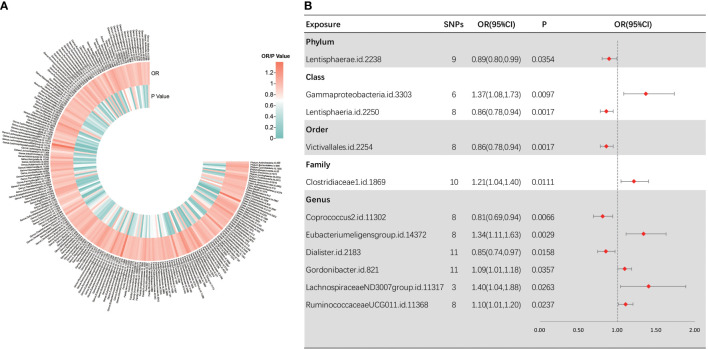
Causal analysis of gut microbiota and sepsis. **(A)** All results of IVW between gut microbiota and sepsis. **(B)** IVW results of gut microbiota taxa with causality to sepsis.

Following several quality control procedures, a total of 2,220 IVs were found to be related to sepsis susceptibility, and 2,219 IVs associated with sepsis (critical care) and sepsis-related death. All *F*-values for inclusion of SNPs >10. For sepsis susceptibility, as shown in **Figure 2**, IVW analysis indicated that 11 bacterial taxa were associated with sepsis susceptibility. Through sensitivity analysis, eight of them fulfilled the criteria for gut microbiota related to sepsis development, including the phylum *Lentisphaerae* [odds ratio (OR) = 0.89, 95% confidence interval (CI), 0.80–0.99, *p* = 0.0354], class *Lentisphaeria* (OR = 0.86, 95% CI, 0.78–0.94, *p* = 0.0017), order *Victivallales* (OR = 0.86, 95% CI, 0.78–0.94, *p* = 0.0017), genus *Dialister* (OR = 0.85, 95% CI, 0.74–0.97, *p* = 0.0158), class *Gammaproteobacteria* (OR = 1.37, 95% CI, 1.08–1.73, *p* = 0.0097), family *Clostridiaceae1* (OR = 1.21, 95% CI, 1.04–1.40, *p* = 0.0111), and genera *RuminococcaceaeUCG01 1* (OR = 1.10, 95% CI, 1.01–1.20, *p* = 0.0237) and *Coprococcus2* (OR = 0.81, 95% CI, 0.69–0.94, *p* = 0.0066).

Among them, the class *Lentisphaeria* and the order *Victivallales* were significantly correlated with a reduced sepsis susceptibility risk. Notably, as the two types of gut microbial taxa are identical, we retained only the result for the order *Victivallales*. Three MR analysis methods showed inconsistency in the direction of the effects of the three bacterial taxa, namely, the genera *Eubacteriumeligensgroup*, *Gordonibacter*, and *LachnospiraceaeND3007group*. In **Supplementary Figure S1**, scatter plots from various tests were presented. Cochran *Q*-derived *p*-values suggested the absence of heterogeneity. Additionally, both the MR-Egger regression and MR-PRESSO tests failed to demonstrate horizontal pleiotropy (all *p* > 0.05) (**Supplementary Table S2**). LOO analyses revealed that individual SNPs do not bias the estimates (**Supplementary Figure S2**). To be clear, MR-PRESSO values and global test could not be measured because there were not enough instrumental variables for the genus *LachnospiraceaeND3007group.*

### Sepsis (critical care) risk

**Figure 3:**
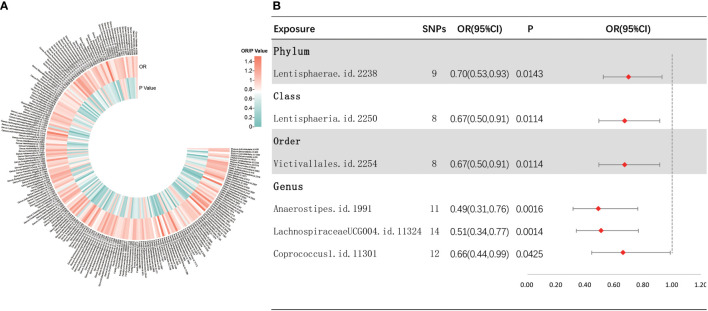
Investigating the causal relationship between gut microbiota and sepsis requiring critical care. **(A)** All results of IVW between gut microbiota and sepsis requiring critical care. **(B)** IVW results of gut microbiota taxa with causality to sepsis requiring critical care.

As depicted in **Figure 3**, the results of IVW analyses revealed that the phylum *Lentisphaerae* (OR = 0.70, 95% CI, 0.53–0.93, *p* = 0.0143), class *Lentisphaeria* (OR = 0.67, 95% CI, 0.50–0.91, *p* = 0.0114), order *Victivallales* (OR = 0.67, 95% CI, 0.50–0.91, *p* = 0.0114), and genera *Anaerostipes* (OR = 0.49, 95% CI, 0.31–0.76, *p* = 0.0016), *LachnospiraceaeUCG004* (OR = 0.51, 95% CI, 0.34–0.77, *p* = 0.0014), and *Coprococcus1* (OR = 0.66, 95% CI, 0.44–0.99, *p* = 0.0425) showed a suggestive negative correlation with sepsis requiring critical care. **Supplementary Figure S3** shows the scatter plots for various tests. A range of sensitivity analyses were conducted, consisting of MR-Egger regression, weighted median, Cochran’s *Q* test, MR-Egger intercept test, MR-PRESSO global test, and LOO analyses to confirm the rigidity of the results presented above (**Supplementary Table S3**, **Supplementary Figure S4**). As the order *Victivallales* and the class *Lentisphaeria* are exactly the same, we only kept the results of the order *Victivallales*.

### Sepsis (28-day death in critical care) risk

**Figure 4:**
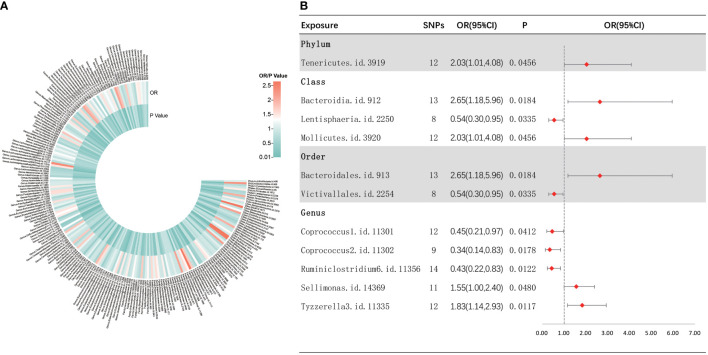
Causal analysis of gut microbiota and sepsis-related 28-day mortality in critical care. **(A)** All results of IVW between gut microbiota and sepsis-related 28-day mortality in critical care. **(B)** IVW results of gut microbiota taxa with causality to sepsis-related 28-day mortality in critical care.

As shown in **Figure 4**, IVW analysis indicated that 11 bacterial taxa were associated with sepsis-related 28-day mortality in critical care. The three MR analysis methods were found to affect two bacterial taxa (i.e., *Sellimonas* and *Tyzzerella3*) in different directions by performing sensitivity analyses (**Supplementary Figure S5**). Finally, nine met the eligibility criteria for gut microbiota in relation to the occurrence of sepsis-related deaths in critical care. Five bacterial taxa were normally significantly negatively related to the risk of sepsis-related death in critical care, including the class *Lentisphaeria* (OR = 0.54, 95% CI, 0.30–0.95, *p* = 0.0335), order *Victivallales* (OR = 0.54, 95% CI, 0.30–0.95, *p* = 0.0335), and genera *Coprococcus2* (OR = 0.34, 95% CI, 0.14–0.83, *p* = 0.0178), *Ruminiclostridium6* (OR = 0.43, 95% CI, 0.22–0.83, *p* = 0.0122), and *Coprococcus1* (OR = 0.45, 95% CI, 0.21–0.97, *p* = 0.0412), while four bacterial taxa showed a normally significant positive association with the risk of death from sepsis in critically ill patients, including the phylum *Tenericutes* (OR = 2.03, 95% CI, 1.01–4.08, *p* = 0.0456), classes *Mollicutes* (OR = 2.03, 95% CI, 1.01–4.08, *p* = 0.0456) and *Bacteroidia* (OR = 2.65, 95% CI, 1.18–5.96, *p* = 0.0289), and order *Bacteroidales* (OR = 2.65, 95% CI, 1.18–5.96, *p* = 0.0184). Furthermore, nine bacterial taxa were subjected to a sequential sensitivity analyses involving MR-Egger regression, weighted median, Cochran’s *Q* test, MR-Egger intercept test, MR-PRESSO global test, and LOO analyses, which confirmed the robustness of the above results (**Supplementary Table S4**, **Supplementary Figure S6**). Interestingly, the order *Victivallales* is identical to the class *Lentisphaeria*, the order *Bacteroidales* is identical to the class *Bacteroidia*, and the class *Mollicutes* is identical to the phylum *Mycoplasmatota*, which is equivalent to *Tenericutes.* Thus, we have retained only the order *Victivallales*, the order *Bacteroidales*, and the class *Mollicutes*.

**Figure 5:**
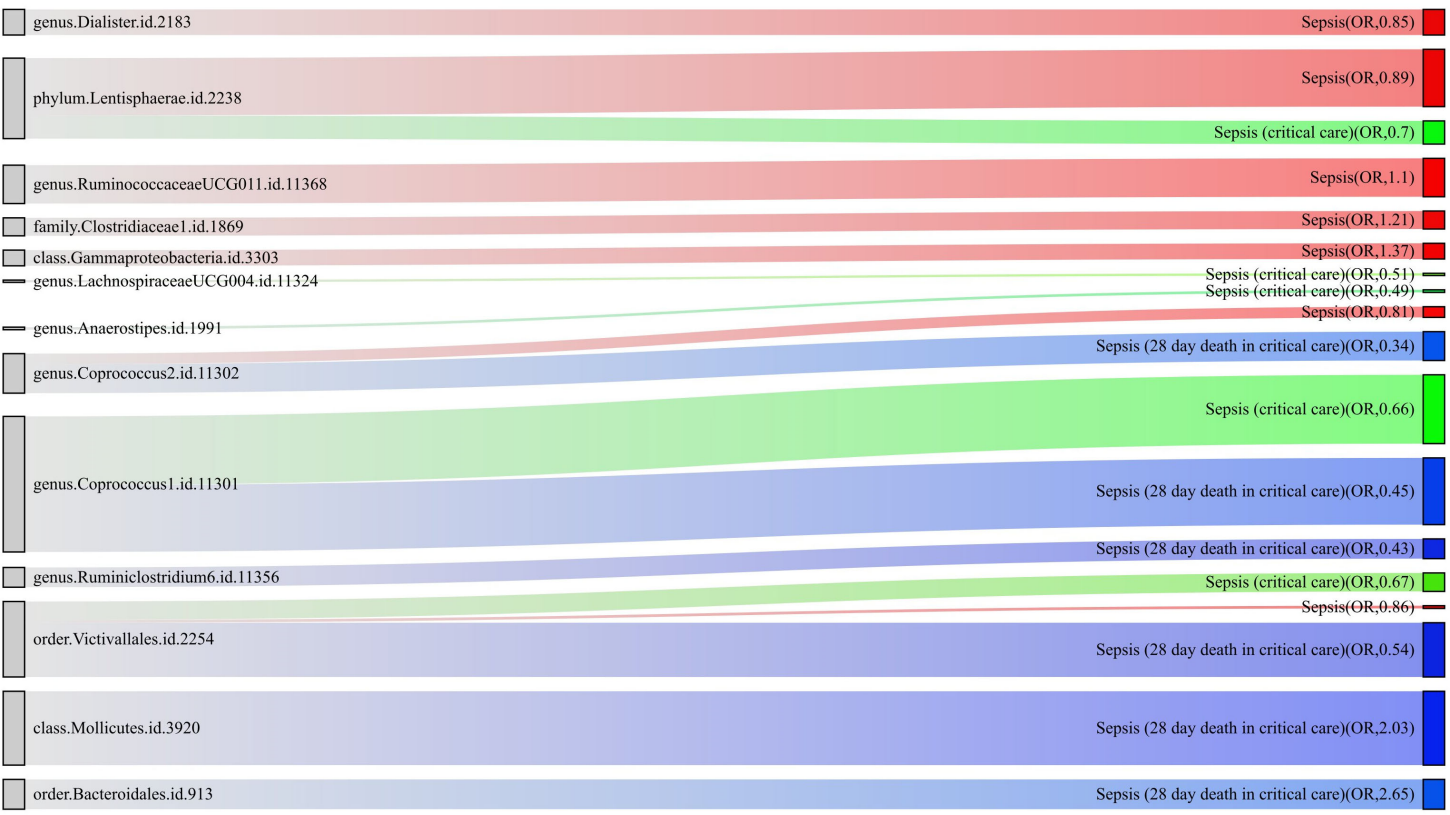
The causality between gut microbiota and sepsis, sepsis requiring critical care, and sepsis-related 28-day mortality in critical care by Mendelian randomization analysis. The thickness of the line represents the *p*-value.

For a better understanding of the causal relationship between gut microbiota and sepsis, a summary network is shown in **Figure 5**.

### Confounding analysis and Steiger test

To assess the impact of confounders, we searched the Phenoscanner V2 website for several of the confounders we identified, including sex, obesity, and diabetes-related instrumental variables.

Regarding sepsis susceptibility, one SNP (rs12636310) of *RuminococcaceaeUCG011* was linked to diabetes-related phenotypes. Causality remained significant after removal of this SNP (IVW OR = 1.11, 95% CI, 1.02–1.22, *p* = 0.0224). Regarding sepsis (critical care) risk, one SNP (rs128942721) for *LachnospiraceaeUCG004* was correlated with obesity-related phenotypes, and the causal relationship remained significant after the removal of one SNP (IVW OR = 0.52, 95% CI, 0.33–0.82, *p* = 0.0045). Regarding sepsis-related 28-day mortality in critical care, SNPs of the bacteria were discovered to be causally related to sepsis-related death, independent of any confounding factors. Further Steiger tests were performed to verify the direction of the gut microbiota influence on sepsis, sepsis requiring critical care, and sepsis (28-day death in critical care). The Steiger *p*-values suggested that established causality is not affected by reverse causality (**Table 1**).

**Table 1:** Steiger direction test from the gut microbiota to sepsis, sepsis (critical care), and sepsis-related 28-day mortality in critical care.

Exposure	Outcome	Direction	Steiger P
phylum *Lentisphaerae*	sepsis	TRUE	5.49E-44
class *Gammaproteobacteria*	sepsis	TRUE	3.22E-28
order *Victivallales*	sepsis	TRUE	6.47E-39
family Clostridiaceae1	sepsis	TRUE	1.60E-44
genus *RuminococcaceaeUCG011*	sepsis	TRUE	1.48E-40
genus *Dialister*	sepsis	TRUE	4.90E-48
genus *Coprococcus2*	sepsis	TRUE	1.15E-34
phylum *Lentisphaerae*	sepsis (critical care)	TRUE	7.82E-44
order *Victivallales*	sepsis (critical care)	TRUE	1.33E-38
genus *LachnospiraceaeUCG004*	sepsis (critical care)	TRUE	1.99E-51
genus *Anaerostipes*	sepsis (critical care)	TRUE	7.02E-48
genus Coprococcus1	sepsis (critical care)	TRUE	1.49E-62
class *Mollicutes*	Sepsis (28-day death in critical care)	TRUE	1.33E-57
order *Victivallales*	Sepsis (28-day death in critical care)	TRUE	6.19E-39
order *Bacteroidales*	Sepsis (28-day death in critical care)	TRUE	1.20E-63
genus *Ruminiclostridium6*	Sepsis (28-day death in critical care)	TRUE	2.49E-66
genus *Coprococcus1*	Sepsis (28-day death in critical care)	TRUE	1.98E-64
genus *Coprococcus2*	Sepsis (28-day death in critical care)	TRUE	1.05E-34

## Discussion

In this research, we adopted a two-sample MR study to explore the causality between gut microbiota and the onset and progression of sepsis. We identified one causal bacterial taxa and six suggestive bacterial taxa associated with the development of sepsis. In addition, five bacterial taxa were suggested to be causally related to sepsis requiring critical care, and six bacterial taxa were suggested to be causally related to sepsis-related 28-day mortality in critical care.

Three bacteria taxa (*RuminococcaceaeUCG011*, *Clostridiaceae1*, and *Gammaproteobacteria*) were positively correlated with susceptibility to sepsis and two bacteria taxa (class *Mollicutes* and order *Bacteroidales*) were positively correlated with sepsis-related mortality. Previous research had shown a higher relative abundance of *Bacteroidetes* and a significantly higher enrichment of *Gammaproteobacteria* in the sepsis group compared to healthy controls, in accordance with our findings (5). Murine studies had also indicated that post-sepsis mice lung communities were clearly enriched with the order *Bacteroidales* found in the murine gut (33). High levels of *Bacteroides* spp. were prevalent in bronchoalveolar lavage fluid from ARDS patients and were significantly correlated with serum TNF-α concentrations, a critical mediator of the septic stress reaction and a predictor of patient mortality (33), while the class *Gammaproteobacteria* included pathogens such as *Escherichia coli* and *Klebsiella* spp., which were normally found in small numbers but have the potential to overgrow and dominate the intestinal tract during dysbiosis (34). The fecal microbiota following injury in a mouse model of sepsis showed that *Ruminococcaceae* increased in the subacute phase (35). *Ruminococcaceae* is a genus in the class *Clostridia*, and *Clostridiaceae* is also a family in the class *Clostridia*. Liu et al. found that the class *Clostridia* was significantly more enriched in the healthy group compared to the sepsis group (5), which contradicts our results. However, their results were limited due to methodological shortcomings (e.g., residual confounding factors). In addition, in the results of our analyses, the genera *RuminococcaceaeUCG011* and *Ruminiclostridium6* had different effects on sepsis risk. The inconsistency of the results may be attributed to the fact that the genus-level classification of the gut microbiota has not been explored in sufficient depth. Research related to *Mollicutes* and sepsis is lacking, but there is some indirect evidence of their relevance. For instance, Sompolinsky et al. found puerperal sepsis caused by T-strain *Mycoplasma* (36), a genus belonging to the class *Mollicutes.*

The MR study found that one causal bacterial taxon (order *Victivallales*) and three suggestive bacterial taxa (genera *Dialister* and *Coprococcus2*, and phylum *Lentisphaerae*) were causally related to sepsis, five bacterial taxa (genera *LachnospiraceaeUCG004*, *Anaerostipes*, and *Coprococcus1*, order *Victivallales*, and phylum *Lentisphaerae*) were suggested to be causally related to sepsis requiring critical care, and four bacterial taxa (genera *Ruminiclostridium6*, *Coprococcus2*, and *Coprococcus1*, and order *Victivallales*) were suggested to be causally related to sepsis-related 28-day mortality in critical care. Interestingly, two species of gut flora (*Coprococcus* and *Victivallales*) have a negative regulatory role in both the development and progression of sepsis. The genera *Coprococcus1*, *Coprococcus2*, *LachnospiraceaeUCG004*, *Anaerostipes*, and *Ruminiclostridium6* all belong to the class *Clostridia*. Children with sepsis had more pathogens of opportunistic origin and fewer beneficial bacteria (e.g., *Clostridia*) detected in their bodies (9). In addition, the adult with sepsis was found to be significantly less enriched in the class *Clostridia* compared to the healthy group (5). The short-chain fatty acid (SCFA) butyrate, generated by *Clostridia*, exerts its effects on colonic regulatory T-cell differentiation by upregulating the key regulatory T-cell transcription factor Foxp3 (37) and suppressing histone deacetylation, thereby reducing the expression of NF-κB-regulated pro-inflammatory cytokines, such as TNF-α and IL-6 (38).

For *Dialister, Victivallales*, and *Lentisphaerae*, very limited investigations have reported their associations with sepsis. *Dialister* was previously found to be closely linked to early-onset neonatal sepsis, but the exact action and its mechanism are not clarified (39), while the order *Victivallales* belongs to the phylum *Lentisphaerae*, which are all Gram-negative bacteria that produce extracellular mucus substances. We hypothesize that this group of bacteria may be involved in the formation of the intestinal mucus barrier and thus play a protective role in the gut (40). Moreover, a reduction in *Lentisphaerae* and others was found in individuals over 65 years, which may affect changes in intestinal physiology such as reduced intestinal contractility, reduced mucus production, intestinal barrier dysfunction, and the ensuing dysbiosis (41).

The gut microbiome may influence host vulnerability and response to sepsis by the following mechanisms. The first mechanism is the expansion of gut pathogenic bacteria. Animal studies have shown that inflammation of the colon and exposure to antibiotics in mice lead to amplification and systemic spread of pathogenic clones of multidrug-resistant *E. coli*, and that the systemic presence of this pathogen is sufficient to induce sepsis-like disease (42). In addition, a single-center cohort study suggested that gut dysbiosis, characterized by an accumulation of *bacilli* and their fermentation metabolites, might precede the onset of late-onset sepsis (43). Second, the immune system produces a powerful pro-inflammatory effect. For instance, in a mouse model of *Streptococcus pneumoniae* sepsis, it was observed that administering oral antibiotics prior to the onset of sepsis was associated with decreased levels of TNF-α (a pro-inflammatory cytokine) in the lungs (44). However, other studies have shown that depletion of the gut microbiota has an increased effect on TNF-α (45, 46). Although the expression of specific cytokines varied across studies, it seems that a stronger inflammatory response to sepsis is the overall effect of altering the structure of the normal gut microbiota prior to the onset of sepsis (47). Third, the production of beneficial microbial products (e.g., SCFAs) is reduced. These metabolites may be involved in the initiation of the innate and adaptive immune system to protect distant organs from infection (48). Fourth, once sepsis is established, the gut microbiome deteriorates and leads to increased susceptibility to visceral organ dysfunction (47). In sum, there is a causality between gut flora and sepsis, which provides a basis for the therapy of sepsis through dietary intervention, the addition of prebiotics, fecal microbiota transplantation (FMT), or the addition of the gut flora metabolite SCFA (47, 49).

This study has the following advantages: MR analysis identified a causal relationship between the gut microbiota and the onset and progression of sepsis, excluding the influence of confounding factors and reverse caution. Genetic variation in the intestinal flora was derived from the largest existing GWAS meta-analysis, and the causal influence of various gut microbiota on sepsis was analyzed from the genus to the phylum level. However, the study suffers from several limitations. First, the majority of the incorporated studies focused on European populations; thus, the findings of this research might not be entirely transferable to other ethnic groups. Second, considering the moderate sample size of the gut microbiota, we opted not to conduct reverse MR analyses, as this approach may be susceptible to potential instrumental biases in the obtained results. Third, 16S rRNA gene sequencing can only depict the gut microbiota at the genus to phylum level; metagenomic and multi-omics approaches might present the possibility of targeting the composition of the gut microbiota at a more concrete level, thus discovering whether a more specific species level is linked to sepsis. Finally, the intestinal microbiota is influenced by a variety of environmental factors such as diet, lifestyle, and medication, and the lack of detailed information in the raw data about disease diet or medication status makes further subgroup analyses difficult.

In summary, the MR study has identified several bacteria that were causally linked to the onset and progression of sepsis, providing new ideas for early diagnosis, personalized treatment, and outcome prognosis of sepsis. However, large samples of population studies as well as *in vivo* and *ex vivo* experimental evidence are still needed to further elucidate the role and mechanisms of specific groups of bacteria in the occurrence and progression of sepsis.

## Data availability statement

Publicly available datasets were analyzed in this study.

## Ethics statement

This study used de-identified data from an open participant study that had been approved by the Ethical Standards Committee for Human Experimentation, so this study did not require separate ethical approval.

## Author contributions

WS: Data curation, Formal analysis, Investigation, Methodology, Software, Validation, Visualization, Writing – original draft. SZ: Formal Analysis, Writing – original draft. HQ: Writing – original draft, Data curation, Software, Visualization. SH: Data curation, Software, Writing – original draft, Investigation. HL: Data curation, Investigation, Software, Writing – original draft. JL: Conceptualization, Supervision, Writing – review & editing. DC: Conceptualization, Supervision, Writing – review & editing.

## Supplementary material

Supplementary Figure 1 Scatter plots for the causality between gut microbiota and sepsis susceptibility.

Supplementary Figure 2 Leave-one-out analyses for the causality between gut microbiota and sepsis susceptibility.

Supplementary Figure 3 Scatter plots for the causality between gut microbiota and sepsis related to critical care.

Supplementary Figure 4 Leave-one-out analyses for the causality between gut microbiota and sepsis related to critical care.

Supplementary Figure 5 Scatter plots for the causality between gut microbiota and sepsis-related 28-day mortality in critical care.

Supplementary Figure 6 Leave-one-out analyses for the causality between gut microbiota and sepsis-related 28-day mortality in critical care.

## References

[1] Evans L Rhodes A Alhazzani W Antonelli M Coopersmith CM French C. Surviving sepsis campaign: international guidelines for management of sepsis and septic shock 2021. Intensive Care Med (2021) 47:1181–247. doi: 10.1007/s00134-021-06506-y PMC8486643 34599691

[2] Minasyan H. Sepsis: mechanisms of bacterial injury to the patient. Scand J Trauma Resusc Emerg Med (2019) 27:19. doi: 10.1186/s13049-019-0596-4 30764843 PMC6376788

[3] Costa SP Carvalho CM. Burden of bacterial bloodstream infections and recent advances for diagnosis. Pathog Dis (2022) 80:ftac027. doi: 10.1093/femspd/ftac027 35790126

[4] Lange K Buerger M Stallmach A Bruns T. Effects of antibiotics on gut microbiota. Dig Dis (2016) 34:260–8. doi: 10.1159/000443360 27028893

[5] Liu Z Li N Fang H Chen X Guo Y Gong S. Enteric dysbiosis is associated with sepsis in patients. FASEB J (2019) 33:12299–310. doi: 10.1096/fj.201900398RR PMC6902702 31465241

[6] Zaborin A Smith D Garfield K Quensen J Shakhsheer B Kade M. Membership and behavior of ultra-low-diversity pathogen communities present in the gut of humans during prolonged critical illness. mBio (2014) 5:e01361–01314. doi: 10.1128/mBio.01361-14 PMC4173762 25249279

[7] Prescott HC Dickson RP Rogers MA Langa KM Iwashyna TJ. Hospitalization type and subsequent severe sepsis. Am J Respir Crit Care Med (2015) 192:581–8. doi: 10.1164/rccm.201503-0483OC PMC4595694 26016947

[8] Liu J Wang M Chen W Ma J Peng Y Zhang M. Altered gut microbiota taxonomic compositions of patients with sepsis in a pediatric intensive care unit. Front Pediatr (2021) 9:645060. doi: 10.3389/fped.2021.645060 33898360 PMC8058355

[9] Du B Shen N Tao Y Sun S Zhang F Ren H. Analysis of gut microbiota alteration and application as an auxiliary prognostic marker for sepsis in children: a pilot study. Transl Pediatr (2021) 10:1647–57. doi: 10.21037/tp-21-51 PMC8261590 34295779

[10] Wan YD Zhu RX Wu ZQ Lyu SY Zhao LX Du ZJ. Gut microbiota disruption in septic shock patients: A pilot study. Med Sci Monit (2018) 24:8639–46. doi: 10.12659/msm.911768 PMC6282651 30488879

[11] Sun S Wang D Dong D Xu L Xie M Wang Y. Altered intestinal microbiome and metabolome correspond to the clinical outcome of sepsis. Crit Care (2023) 27:127. doi: 10.1186/s13054-023-04412-x 36978107 PMC10044080

[12] Panpetch W Somboonna N Bulan DE Issara-Amphorn J Worasilchai N Finkelman M. Gastrointestinal colonization of candida albicans increases serum (1→3)-β-D-glucan, without candidemia, and worsens cecal ligation and puncture sepsis in murine model. Shock (2018) 49:62–70. doi: 10.1097/shk.0000000000000896 28498297

[13] Chen G Huang B Fu S Li B Ran X He D. G protein-coupled receptor 109A and host microbiota modulate intestinal epithelial integrity during sepsis. Front Immunol (2018) 9:2079. doi: 10.3389/fimmu.2018.02079 30271409 PMC6146091

[14] Smith GD Ebrahim S. 'Mendelian randomization': can genetic epidemiology contribute to understanding environmental determinants of disease? Int J Epidemiol (2003) 32:1–22. doi: 10.1093/ije/dyg070 12689998

[15] Chen JH Zeng LY Zhao YF Tang HX Lei H Wan YF. Causal effects of gut microbiota on sepsis: a two-sample Mendelian randomization study. Front Microbiol (2023) 14:1167416. doi: 10.3389/fmicb.2023.1167416 37234519 PMC10206031

[16] Zhang Z Cheng L Ning D. Gut microbiota and sepsis: bidirectional Mendelian study and mediation analysis. Front Immunol (2023) 14:1234924. doi: 10.3389/fimmu.2023.1234924 37662942 PMC10470830

[17] Boef AG Dekkers OM le Cessie S. Mendelian randomization studies: a review of the approaches used and the quality of reporting. Int J Epidemiol (2015) 44:496–511. doi: 10.1093/ije/dyv071 25953784

[18] Kurilshikov A Medina-Gomez C Bacigalupe R Radjabzadeh D Wang J Demirkan A. Large-scale association analyses identify host factors influencing human gut microbiome composition. Nat Genet (2021) 53:156–65. doi: 10.1038/s41588-020-00763-1 PMC8515199 33462485

[19] Bycroft C Freeman C Petkova D Band G Elliott LT Sharp K. The UK Biobank resource with deep phenotyping and genomic data. Nature (2018) 562:203–9. doi: 10.1038/s41586-018-0579-z PMC6786975 30305743

[20] Zekavat SM Lin SH Bick AG Liu A Paruchuri K Wang C. Hematopoietic mosaic chromosomal alterations increase the risk for diverse types of infection. Nat Med (2021) 27:1012–24. doi: 10.1038/s41591-021-01371-0 PMC8245201 34099924

[21] Hamilton FW Thomas M Arnold D Palmer T Moran E Mentzer AJ. Therapeutic potential of IL6R blockade for the treatment of sepsis and sepsis-related death: A Mendelian randomisation study. PloS Med (2023) 20:e1004174. doi: 10.1371/journal.pmed.1004174 36716318 PMC9925069

[22] Ni JJ Xu Q Yan SS Han BX Zhang H Wei XT. Gut microbiota and psychiatric disorders: A two-sample mendelian randomization study. Front Microbiol (2021) 12:737197. doi: 10.3389/fmicb.2021.737197 35185808 PMC8856606

[23] Pierce BL Ahsan H Vanderweele TJ. Power and instrument strength requirements for Mendelian randomization studies using multiple genetic variants. Int J Epidemiol (2011) 40:740–52. doi: 10.1093/ije/dyq151 PMC3147064 20813862

[24] Pierce BL Burgess S. Efficient design for Mendelian randomization studies: subsample and 2-sample instrumental variable estimators. Am J Epidemiol (2013) 178:1177–84. doi: 10.1093/aje/kwt084 PMC3783091 23863760

[25] Bowden J Davey Smith G Burgess S. Mendelian randomization with invalid instruments: effect estimation and bias detection through Egger regression. Int J Epidemiol (2015) 44:512–25. doi: 10.1093/ije/dyv080 PMC4469799 26050253

[26] Bowden J Davey Smith G Haycock PC Burgess S. Consistent estimation in mendelian randomization with some invalid instruments using a weighted median estimator. Genet Epidemiol (2016) 40:304–14. doi: 10.1002/gepi.21965 PMC4849733 27061298

[27] Verbanck M Chen CY Neale B Do R. Detection of widespread horizontal pleiotropy in causal relationships inferred from Mendelian randomization between complex traits and diseases. Nat Genet (2018) 50:693–8. doi: 10.1038/s41588-018-0099-7 PMC6083837 29686387

[28] Thompson KJ Finfer SR Woodward M Leong RNF Liu B. Sex differences in sepsis hospitalisations and outcomes in older women and men: A prospective cohort study. J Infect (2022) 84:770–6. doi: 10.1016/j.jinf.2022.04.035 35472366

[29] Wang HE Griffin R Judd S Shapiro NI Safford MM. Obesity and risk of sepsis: a population-based cohort study. Obes (Silver Spring) (2013) 21:E762–769. doi: 10.1002/oby.20468 PMC3795990 23526732

[30] Koh GC Peacock SJ van der Poll T Wiersinga WJ. The impact of diabetes on the pathogenesis of sepsis. Eur J Clin Microbiol Infect Dis (2012) 31:379–88. doi: 10.1007/s10096-011-1337-4 PMC3303037 21805196

[31] Hemani G Tilling K Davey Smith G. Orienting the causal relationship between imprecisely measured traits using GWAS summary data. PloS Genet (2017) 13:e1007081. doi: 10.1371/journal.pgen.1007081 29149188 PMC5711033

[32] Skrivankova VW Richmond RC Woolf BAR Davies NM Swanson SA VanderWeele TJ. Strengthening the reporting of observational studies in epidemiology using mendelian randomisation (STROBE-MR): explanation and elaboration. Bmj (2021) 375:n2233. doi: 10.1136/bmj.n2233 34702754 PMC8546498

[33] Dickson RP Singer BH Newstead MW Falkowski NR Erb-Downward JR Standiford TJ. Enrichment of the lung microbiome with gut bacteria in sepsis and the acute respiratory distress syndrome. Nat Microbiol (2016) 1:16113. doi: 10.1038/nmicrobiol.2016.113 27670109 PMC5076472

[34] Kim S Covington A Pamer EG. The intestinal microbiota: Antibiotics, colonization resistance, and enteric pathogens. Immunol Rev (2017) 279:90–105. doi: 10.1111/imr.12563 28856737 PMC6026851

[35] Muratsu A Ikeda M Shimizu K Kameoka S Motooka D Nakamura S. Dynamic change of fecal microbiota and metabolomics in a polymicrobial murine sepsis model. Acute Med Surg (2022) 9:e770. doi: 10.1002/ams2.770 35782956 PMC9238289

[36] Sompolinsky D Solomon F Leiba H Caspi E Lewinsohn G Almog C. Puerperal sepsis due to T-strain Mycoplasma. Isr J Med Sci (1971) 7:745–8. 5560012

[37] Furusawa Y Obata Y Fukuda S TA E Nakato G Takahashi D. Commensal microbe-derived butyrate induces the differentiation of colonic regulatory T cells. Nature (2013) 504:446–50. doi: 10.1038/nature12721 24226770

[38] Parada Venegas D de la Fuente MK Landskron G González MJ Quera R Dijkstra G. Short chain fatty acids (SCFAs)-mediated gut epithelial and immune regulation and its relevance for inflammatory bowel diseases. Front Immunol (2019) 10:277. doi: 10.3389/fimmu.2019.00277 30915065 PMC6421268

[39] Dornelles LV Procianoy RS Roesch LFW Corso AL Dobbler PT Mai V. Meconium microbiota predicts clinical early-onset neonatal sepsis in preterm neonates. J Matern Fetal Neonatal Med (2022) 35:1935–43. doi: 10.1080/14767058.2020.1774870 32508165

[40] Cho JC Vergin KL Morris RM Giovannoni SJ. Lentisphaera araneosa gen. nov., sp. nov, a transparent exopolymer producing marine bacterium, and the description of a novel bacterial phylum, Lentisphaerae. Environ Microbiol (2004) 6:611–21. doi: 10.1111/j.1462-2920.2004.00614.x 15142250

[41] Kundu P Blacher E Elinav E Pettersson S. Our gut microbiome: the evolving inner self. Cell (2017) 171:1481–93. doi: 10.1016/j.cell.2017.11.024 29245010

[42] Ayres JS Trinidad NJ Vance RE. Lethal inflammasome activation by a multidrug-resistant pathobiont upon antibiotic disruption of the microbiota. Nat Med (2012) 18:799–806. doi: 10.1038/nm.2729 22522562 PMC3472005

[43] Graspeuntner S WasChina S Künzel S Twisselmann N Rausch TK Cloppenborg-Schmidt K. Gut dysbiosis with bacilli dominance and accumulation of fermentation products precedes late-onset sepsis in preterm infants. Clin Infect Dis (2019) 69:268–77. doi: 10.1093/cid/ciy882 30329017

[44] Schuijt TJ Lankelma JM Scicluna BP de Sousa e Melo F Roelofs JJ de Boer JD. The gut microbiota plays a protective role in the host defence against pneumococcal pneumonia. Gut (2016) 65:575–83. doi: 10.1136/gutjnl-2015-309728 PMC4819612 26511795

[45] Ikeda M Shimizu K Ogura H Kurakawa T Umemoto E Motooka D. Hydrogen-rich saline regulates intestinal barrier dysfunction, dysbiosis, and bacterial translocation in a murine model of sepsis. Shock (2018) 50:640–7. doi: 10.1097/shk.0000000000001098 29293174

[46] Morowitz MJ Di Caro V Pang D Cummings J Firek B Rogers MB. Dietary supplementation with nonfermentable fiber alters the gut microbiota and confers protection in murine models of sepsis. Crit Care Med (2017) 45:e516–23. doi: 10.1097/ccm.0000000000002291 PMC5392159 28252538

[47] Adelman MW Woodworth MH Langelier C Busch LM Kempker JA Kraft CS. The gut microbiome's role in the development, maintenance, and outcomes of sepsis. Crit Care (2020) 24:278. doi: 10.1186/s13054-020-02989-1 32487252 PMC7266132

[48] Biemond JJ McDonald B Haak BW. Leveraging the microbiome in the treatment of sepsis: potential pitfalls and new perspectives. Curr Opin Crit Care (2023) 29:123–9. doi: 10.1097/mcc.0000000000001019 36762681

[49] Haak BW Prescott HC Wiersinga WJ. Therapeutic potential of the gut microbiota in the prevention and treatment of sepsis. Front Immunol (2018) 9:2042. doi: 10.3389/fimmu.2018.02042 30250472 PMC6139316

