# Supplementary figures and images for: Gut microbiota and sepsis and sepsis-related death: a Mendelian randomization investigation
Source: Front Immunol. 2024 Jan 31;15:1266230. doi: 10.3389/fimmu.2024.1266230 (PMC10867964; doi:10.3389/fimmu.2024.1266230)

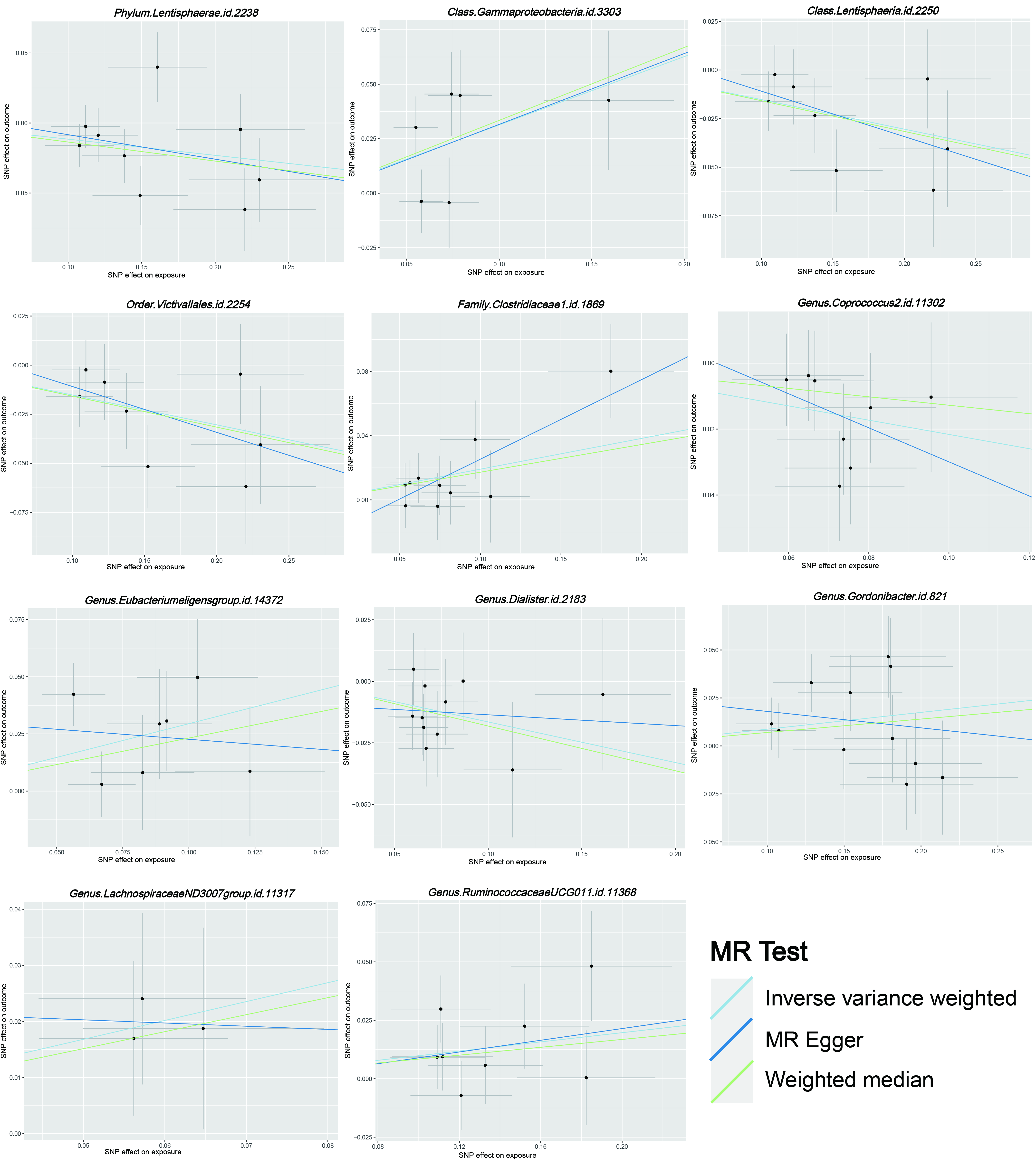

Supplement: Supplementary Figure 1 — Scatter plots for the causality between gut microbiota and sepsis susceptibility. [file Image_1.tif]

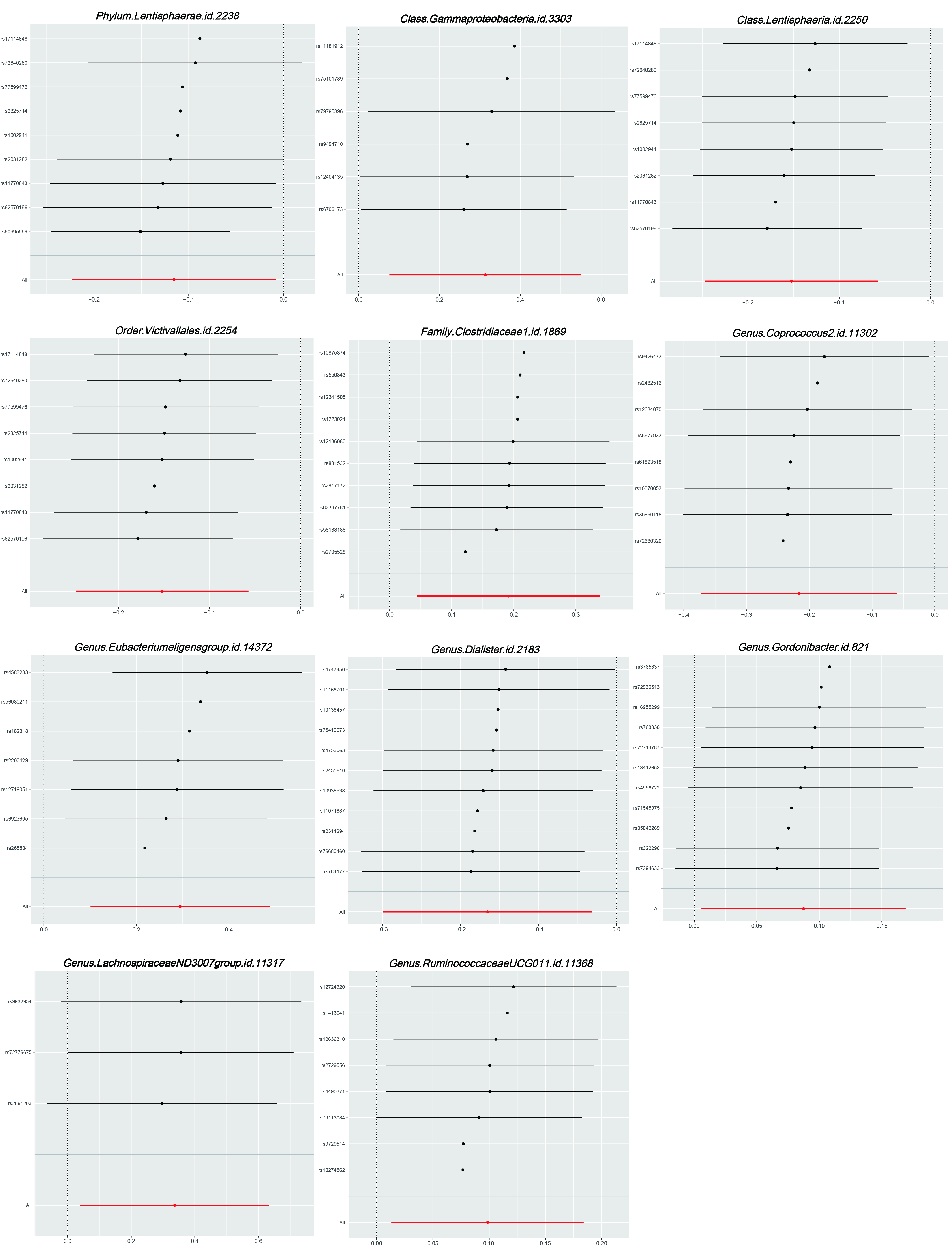

Supplement: Supplementary Figure 2 — Leave-one-out analyses for the causality between gut microbiota and sepsis susceptibility. [file Image_2.tif]

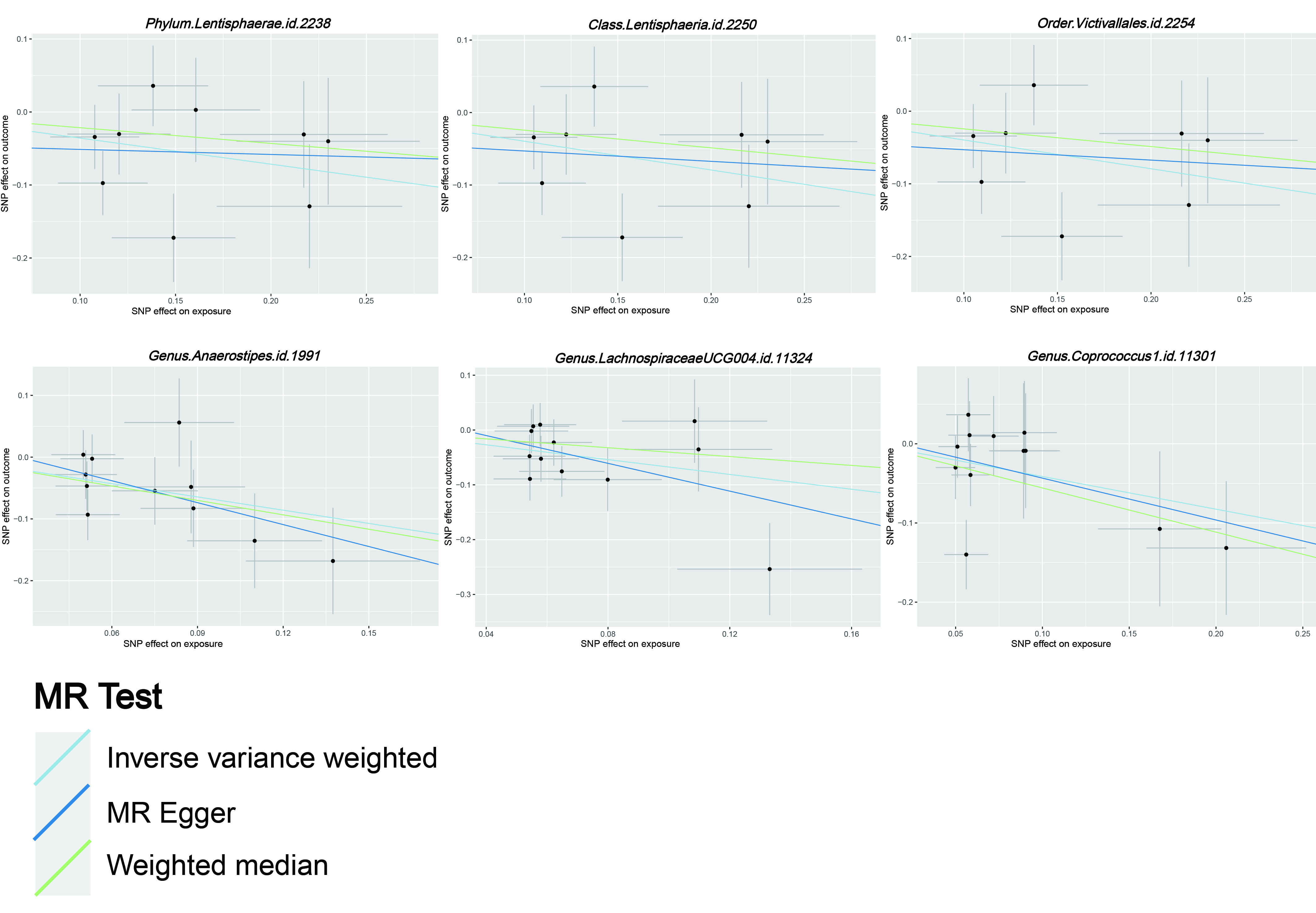

Supplement: Supplementary Figure 3 — Scatter plots for the causality between gut microbiota and sepsis related to critical care. [file Image_3.tif]

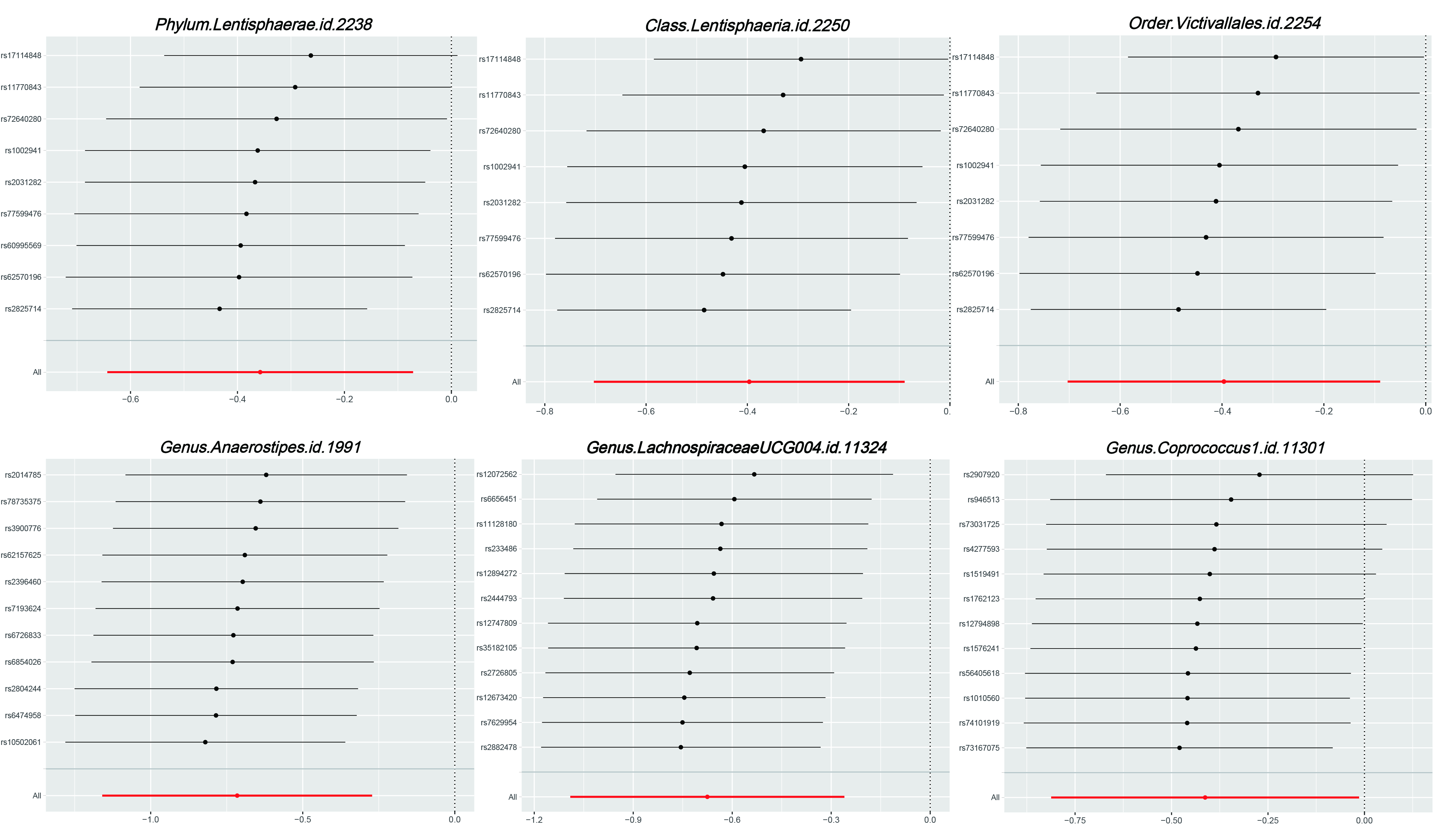

Supplement: Supplementary Figure 4 — Leave-one-out analyses for the causality between gut microbiota and sepsis related to critical care. [file Image_4.tif]

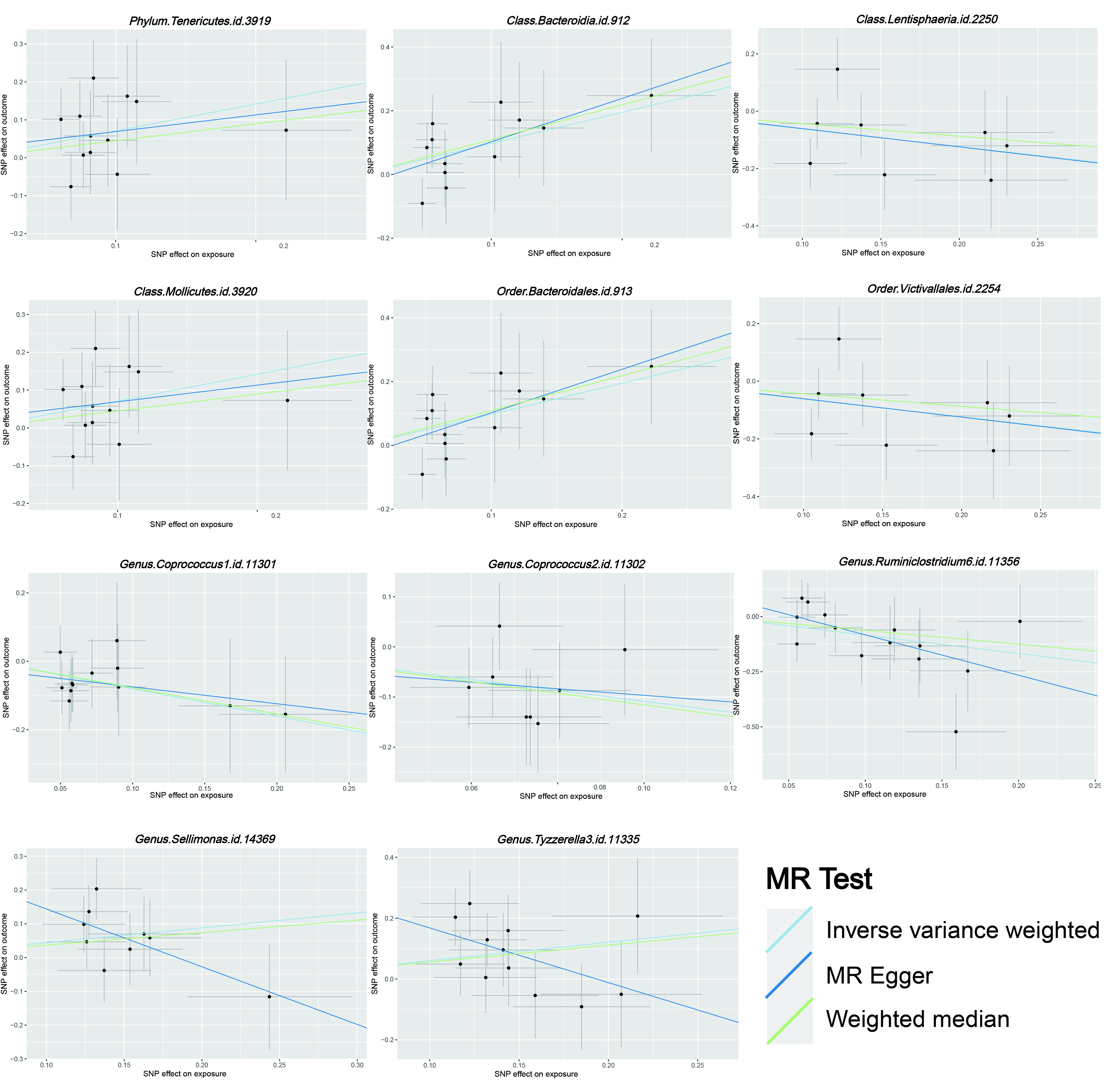

Supplement: Supplementary Figure 5 — Scatter plots for the causality between gut microbiota and sepsis-related 28-day mortality in critical care. [file Image_5.tif]

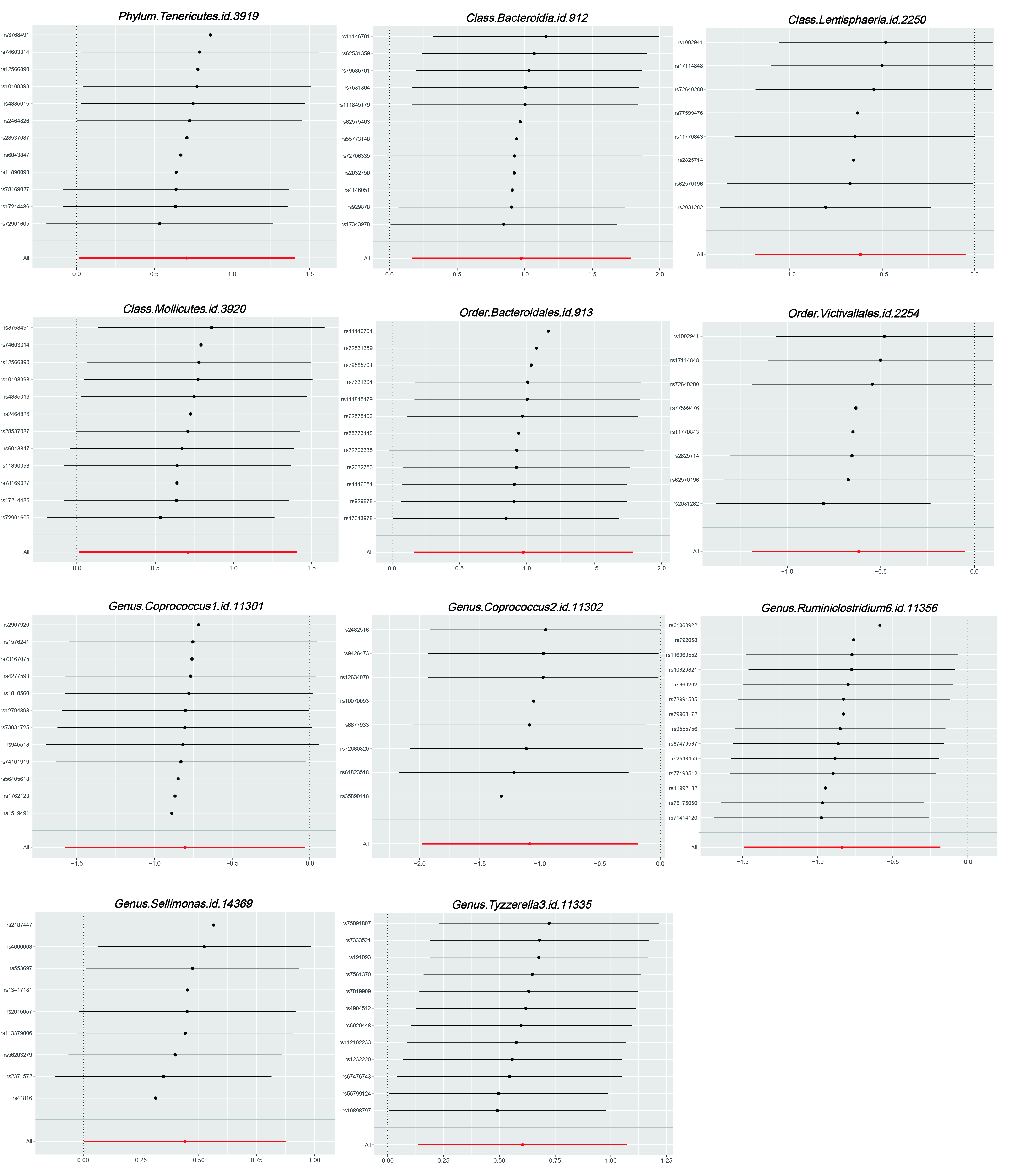

Supplement: Supplementary Figure 6 — Leave-one-out analyses for the causality between gut microbiota and sepsis-related 28-day mortality in critical care. [file Image_6.tif]
