# Supplementary material for: Gut microbiota and sepsis and sepsis-related death: a Mendelian randomization investigation
Source: Front Immunol. 2024 Jan 31;15:1266230. doi: 10.3389/fimmu.2024.1266230 (PMC10867964; doi:10.3389/fimmu.2024.1266230)
Supplement: Supplementary file 8 [file Table_2.docx]

| **Table S2 Sensitivity analysis results for the causal association between gut microbiota and sepsis susceptibility.** | | | | | | | | | |
| --- | --- | --- | --- | --- | --- | --- | --- | --- | --- |
| Exposure | Method | SNPs | Beta | P | OR | 95%CI | Heterogeneity | Pleiotropy | Global test |
|  |  |  |  |  |  |  | P | egger_intercept_p | P |
| phylum.Lentisphaerae | IVW | 9 | -0.1154 | 0.0354 | 0.89 | （0.80，0.99） | 0.1821 | 0.7810 | 0.2276 |
| phylum.Lentisphaerae | MR Egger | 9 | -0.1748 | 0.4399 | 0.84 | （0.55，1.28） | 0.1290 |  |  |
| phylum.Lentisphaerae | WM | 9 | -0.1360 | 0.0407 | 0.87 | （0.77，0.99） |  |  |  |
| class.Gammaproteobacteria | IVW | 6 | 0.3133 | 0.0097 | 1.37 | （1.08，1.73） | 0.2113 | 0.9786 | 0.3008 |
| class.Gammaproteobacteria | MR Egger | 6 | 0.3248 | 0.4872 | 1.38 | （0.60，3.18） | 0.1294 |  |  |
| class.Gammaproteobacteria | WM | 6 | 0.3344 | 0.0246 | 1.40 | （1.04，1.87） |  |  |  |
| class.Lentisphaeria | IVW | 8 | -0.1522 | 0.0017 | 0.86 | （0.78，0.94） | 0.6406 | 0.6282 | 0.6732 |
| class.Lentisphaeria | MR Egger | 8 | -0.2338 | 0.2113 | 0.79 | （0.57，1.10） | 0.5569 |  |  |
| class.Lentisphaeria | WM | 8 | -0.1580 | 0.0163 | 0.85 | （0.75，0.97） |  |  |  |
| order.Victivallales | IVW | 8 | -0.1522 | 0.0017 | 0.86 | （0.78，0.94） | 0.6406 | 0.6282 | 0.6626 |
| order.Victivallales | MR Egger | 8 | -0.2338 | 0.2113 | 0.79 | （0.57，1.10） | 0.5569 |  |  |
| order.Victivallales | WM | 8 | -0.1580 | 0.0169 | 0.85 | （0.75，0.97） |  |  |  |
| family.Clostridiaceae1 | IVW | 10 | 0.1916 | 0.0111 | 1.21 | （1.04，1.40） | 0.7762 | 0.1684 | 0.7388 |
| family.Clostridiaceae1 | MR Egger | 10 | 0.4959 | 0.0496 | 1.64 | （1.08，2.50） | 0.9114 |  |  |
| family.Clostridiaceae1 | WM | 10 | 0.1726 | 0.0968 | 1.19 | （0.97，1.46） |  |  |  |
| genus.Coprococcus2 | IVW | 8 | -0.2162 | 0.0066 | 0.81 | （0.69, 0.94） | 0.7630 | 0.6454 | 0.7872 |
| genus.Coprococcus2 | MR Egger | 8 | -0.5164 | 0.4403 | 0.60 | （0.18, 2.03） | 0.6889 |  |  |
| genus.Coprococcus2 | WM | 8 | -0.1277 | 0.2249 | 0.88 | （0.72, 1.08） |  |  |  |
| genus.Eubacteriumeligensgroup | IVW | 7 | 0.2948 | 0.0029 | 1.34 | （1.11，1.63） | 0.3395 | 0.3673 | 0.3798 |
| genus.Eubacteriumeligensgroup | MR Egger | 7 | -0.0879 | 0.8343 | 0.92 | （0.42，2.00） | 0.3380 |  |  |
| genus.Eubacteriumeligensgroup | WM | 7 | 0.2320 | 0.0805 | 1.26 | （0.97，1.64） |  |  |  |
| genus.Dialister | IVW | 11 | -0.1649 | 0.0158 | 0.85 | （0.74，0.97） | 0.9090 | 0.6582 | 0.9146 |
| genus.Dialister | MR Egger | 11 | -0.0438 | 0.8762 | 0.96 | （0.56，1.64） | 0.8746 |  |  |
| genus.Dialister | WM | 11 | -0.1816 | 0.0390 | 0.83 | （0.70，0.99） |  |  |  |
| genus.Gordonibacter | IVW | 11 | 0.0874 | 0.0357 | 1.09 | （1.01，1.18） | 0.3257 | 0.3316 | 0.352 |
| genus.Gordonibacter | MR Egger | 11 | -0.0856 | 0.6337 | 0.92 | （0.65，1.29） | 0.3326 |  |  |
| genus.Gordonibacter | WM | 11 | 0.0701 | 0.2196 | 1.07 | （0.96，1.20） |  |  |  |
| genus.LachnospiraceaeND3007group | IVW | 3 | 0.3364 | 0.0263 | 1.40 | （1.04，1.88） | 0.9294 | 0.9023 | NA |
| genus.LachnospiraceaeND3007group | MR Egger | 3 | -0.0570 | 0.9857 | 0.94 | （0.01，138.98） | 0.7264 |  |  |
| genus.LachnospiraceaeND3007group | WM | 3 | 0.3031 | 0.1102 | 1.35 | （0.93，1.96） |  |  |  |
| genus.RuminococcaceaeUCG011 | IVW | 8 | 0.0985 | 0.0237 | 1.10 | （1.01，1.20） | 0.5195 | 0.9089 | 0.5534 |
| genus.RuminococcaceaeUCG011 | MR Egger | 8 | 0.1253 | 0.6032 | 1.13 | （0.72，1.77） | 0.4055 |  |  |
| genus.RuminococcaceaeUCG011 | WM | 8 | 0.0841 | 0.1633 | 1.09 | （0.97，1.22） |  |  |  |

IVW, Inverse variance weighted; WM,Weighted median.
