# Supplementary material for: Gut microbiota and sepsis and sepsis-related death: a Mendelian randomization investigation
Source: Front Immunol. 2024 Jan 31;15:1266230. doi: 10.3389/fimmu.2024.1266230 (PMC10867964; doi:10.3389/fimmu.2024.1266230)
Supplement: Supplementary file 9 [file Table_3.docx]

| **Table S3 Sensitivity analysis results for the causal association between gut microbiota and sepsis related to critical care admission.** | | | | | | | | | |
| --- | --- | --- | --- | --- | --- | --- | --- | --- | --- |
| Exposure | Method | SNPs | Beta | P | OR | 95%CI | Heterogeneity | Pleiotropy | Global test |
|  |  |  |  |  |  |  | P | egger_intercept_p | P |
| phylum.Lentisphaerae | IVW | 9 | -0.3575 | 0.0143 | 0.70 | (0.53,0.93) | 0.2842 | 0.6099 | 0.3354 |
| phylum.Lentisphaerae | MR Egger | 9 | -0.0696 | 0.9046 | 0.93 | (0.31,2.80) | 0.2283 |  |  |
| phylum.Lentisphaerae | WM | 9 | -0.2152 | 0.2360 | 0.81 | (0.56,1.15) |  |  |  |
| class.Lentisphaeria | IVW | 8 | -0.3963 | 0.0114 | 0.67 | (0.50,0.91) | 0.2601 | 0.6616 | 0.3144 |
| class.Lentisphaeria | MR Egger | 8 | -0.1430 | 0.8119 | 0.87 | (0.28,2.68) | 0.1977 |  |  |
| class.Lentisphaeria | WM | 8 | -0.2436 | 0.2289 | 0.78 | (0.53,1.17) |  |  |  |
| order.Victivallales | IVW | 8 | -0.3963 | 0.0114 | 0.67 | (0.50,0.91) | 0.2601 | 0.6616 | 0.3124 |
| order.Victivallales | MR Egger | 8 | -0.1430 | 0.8119 | 0.87 | (0.28,2.68) | 0.1977 |  |  |
| order.Victivallales | WM | 8 | -0.2436 | 0.2101 | 0.78 | (0.54,1.15) |  |  |  |
| genus.Anaerostipes | IVW | 11 | -0.7156 | 0.0016 | 0.49 | (0.31,0.76) | 0.6534 | 0.5072 | 0.6896 |
| genus.Anaerostipes | MR Egger | 11 | -1.1835 | 0.1319 | 0.31 | (0.08,1.24) | 0.6089 |  |  |
| genus.Anaerostipes | WM | 11 | -0.7782 | 0.0100 | 0.46 | (0.25,0.83) |  |  |  |
| genus.LachnospiraceaeUCG004 | IVW | 12 | -0.6754 | 0.0014 | 0.51 | (0.34,0.77) | 0.3654 | 0.4673 | 0.4062 |
| genus.LachnospiraceaeUCG004 | MR Egger | 12 | -1.2677 | 0.1501 | 0.28 | (0.06,1.39) | 0.3324 |  |  |
| genus.LachnospiraceaeUCG004 | WM | 12 | -0.4039 | 0.1764 | 0.67 | (0.37,1.20) |  |  |  |
| genus.Coprococcus1 | IVW | 12 | -0.4129 | 0.0425 | 0.66 | (0.44,0.99) | 0.3782 | 0.8051 | 0.4294 |
| genus.Coprococcus1 | MR Egger | 12 | -0.5290 | 0.3194 | 0.59 | (0.22,1.58) | 0.3033 |  |  |
| genus.Coprococcus1 | WM | 12 | -0.5579 | 0.0345 | 0.57 | (0.34,0.96) |  |  |  |

IVW, Inverse variance weighted; WM,Weighted median.
