# Supplementary material for: Gut microbiota and sepsis and sepsis-related death: a Mendelian randomization investigation
Source: Front Immunol. 2024 Jan 31;15:1266230. doi: 10.3389/fimmu.2024.1266230 (PMC10867964; doi:10.3389/fimmu.2024.1266230)
Supplement: Supplementary file 10 [file Table_4.docx]

| **Table S4 Sensitivity analysis results for the causal association between gut microbiota and sepsis related to death.** | | | | | | | | | |
| --- | --- | --- | --- | --- | --- | --- | --- | --- | --- |
| Exposure | Method | SNPs | Beta | P | OR | 95%CI | Heterogeneity | Pleiotropy | Global test |
|  |  |  |  |  |  |  | P | egger_intercept_p | P |
| phylum.Tenericutes | IVW | 12 | 0.7094 | 0.0456 | 2.03 | (1.01,4.08) | 0.7956 | 0.8086 | 0.7984 |
| phylum.Tenericutes | MR Egger | 12 | 0.4403 | 0.7071 | 1.55 | (0.17,14.47) | 0.7273 |  |  |
| phylum.Tenericutes | WM | 12 | 0.4485 | 0.3362 | 1.57 | (0.63,3.91) |  |  |  |
| class.Bacteroidia | IVW | 12 | 0.9747 | 0.0184 | 2.65 | (1.18,5.96) | 0.8240 | 0.6390 | 0.8538 |
| class.Bacteroidia | MR Egger | 12 | 1.3625 | 0.1619 | 3.91 | (0.67,22.89) | 0.7761 |  |  |
| class.Bacteroidia | WM | 12 | 1.0934 | 0.0623 | 2.98 | (0.95,9.42) |  |  |  |
| class.Lentisphaeria | IVW | 8 | -0.6181 | 0.0335 | 0.54 | (0.30,0.95) | 0.3651 | 0.9852 | 0.4224 |
| class.Lentisphaeria | MR Egger | 8 | -0.6381 | 0.5780 | 0.53 | (0.06,4.43) | 0.2654 |  |  |
| class.Lentisphaeria | WM | 8 | -0.4351 | 0.2434 | 0.65 | (0.31,1.34) |  |  |  |
| class.Mollicutes | IVW | 12 | 0.7094 | 0.0456 | 2.03 | (1.01,4.08) | 0.7956 | 0.8086 | 0.8048 |
| class.Mollicutes | MR Egger | 12 | 0.4403 | 0.7071 | 1.55 | (0.17,14.47) | 0.7273 |  |  |
| class.Mollicutes | WM | 12 | 0.4485 | 0.3441 | 1.57 | (0.62,3.97) |  |  |  |
| order.Bacteroidales | IVW | 12 | 0.9747 | 0.0184 | 2.65 | (1.18,5.96) | 0.8240 | 0.6390 | 0.8542 |
| order.Bacteroidales | MR Egger | 12 | 1.3625 | 0.1619 | 3.91 | (0.67,22.89) | 0.7761 |  |  |
| order.Bacteroidales | WM | 12 | 1.0934 | 0.0584 | 2.98 | (0.96,9.26) |  |  |  |
| order.Victivallales | IVW | 8 | -0.6181 | 0.0335 | 0.54 | (0.30,0.95) | 0.3651 | 0.9852 | 0.4178 |
| order.Victivallales | MR Egger | 8 | -0.6381 | 0.5780 | 0.53 | (0.06,4.43) | 0.2654 |  |  |
| order.Victivallales | WM | 8 | -0.4351 | 0.2373 | 0.65 | (0.31,1.33) |  |  |  |
| genus.Coprococcus1 | IVW | 12 | -0.8031 | 0.0412 | 0.45 | (0.21,0.97) | 0.9943 | 0.7224 | 0.9946 |
| genus.Coprococcus1 | MR Egger | 12 | -0.4939 | 0.6080 | 0.61 | (0.10,3.80) | 0.9901 |  |  |
| genus.Coprococcus1 | WM | 12 | -0.7712 | 0.1429 | 0.46 | (0.16,1.30) |  |  |  |
| genus.Coprococcus2 | IVW | 8 | -1.0863 | 0.0178 | 0.34 | (0.14,0.83) | 0.8224 | 0.9080 | 0.8488 |
| genus.Coprococcus2 | MR Egger | 8 | -0.6552 | 0.8617 | 0.52 | (0.00,607.03) | 0.7300 |  |  |
| genus.Coprococcus2 | WM | 8 | -1.1577 | 0.0462 | 0.31 | (0.10,0.98) |  |  |  |
| genus.Ruminiclostridium6 | IVW | 14 | -0.8384 | 0.0122 | 0.43 | (0.22,0.83) | 0.4533 | 0.1971 | 0.4698 |
| genus.Ruminiclostridium6 | MR Egger | 14 | -1.8301 | 0.0410 | 0.16 | (0.03,0.77) | 0.5234 |  |  |
| genus.Ruminiclostridium6 | WM | 14 | -0.6251 | 0.1971 | 0.54 | (0.21,1.38) |  |  |  |
| genus.Sellimonas | IVW | 9 | 0.4400 | 0.0480 | 1.55 | (1.00,2.40) | 0.5228 | 0.1224 | 0.5452 |
| genus.Sellimonas | MR Egger | 9 | -1.7087 | 0.2117 | 0.18 | (0.02,2.07) | 0.7748 |  |  |
| genus.Sellimonas | WM | 9 | 0.3690 | 0.2181 | 1.45 | (0.80,2.60) |  |  |  |
| genus.Tyzzerella3 | IVW | 12 | 0.6042 | 0.0117 | 1.83 | (1.14,2.93) | 0.5073 | 0.0889 | 0.5372 |
| genus.Tyzzerella3 | MR Egger | 12 | -1.7994 | 0.1958 | 0.17 | (0.01,2.11) | 0.7526 |  |  |
| genus.Tyzzerella3 | WM | 12 | 0.5569 | 0.0920 | 1.75 | (0.91,3.34) |  |  |  |

IVW, Inverse variance weighted; WM,Weighted median.
